# The situation of former adolescent self-injurers as young adults: a follow-up study

**DOI:** 10.1186/s12888-015-0555-1

**Published:** 2015-07-18

**Authors:** Rebecca C. Groschwitz, Paul L. Plener, Michael Kaess, Teresa Schumacher, Ramona Stoehr, Isabel Boege

**Affiliations:** Department of Child and Adolescent Psychiatry and Psychotherapy, University of Ulm, Steinhoevelstr. 5, 89075 Ulm, Germany; Department of Child and Adolescent Psychiatry, Centre for Psychosocial Medicine, University Hospital Heidelberg, Blumenstr. 8, 69115 Heidelberg, Germany; Department of Child and Adolescent Psychiatry Weissenau, Centre for Psychiatry in South-Württemberg, Weingartshofer Straße 2, 88214 Ravensburg-Weissenau, Germany

**Keywords:** Non-suicidal self-injury, NSSI, Longitudinal, Follow-up

## Abstract

**Background:**

Nonsuicidal self-injury (NSSI) in adolescence has been described as comorbid condition in affective or anxiety disorders, as well as borderline personality disorder (BPD) and is a risk factor for later suicide attempts. Prevalence rates of NSSI decline steeply from adolescence to young adulthood. Yet, to the best of our knowledge, the longitudinal development of adolescent psychiatric patients with NSSI into their young adulthood has not been investigated. The aim of this study was to assess current NSSI and psychological impairment of young adults, who had been in treatment for NSSI in their adolescence.

**Methods:**

Former patients of the departments of child and adolescent psychiatry and psychotherapy in Ulm and Ravensburg, Germany (*N* = 52), who presented with NSSI in their adolescence, were recruited (average age: 21.5 years (SD = 2.6)). Data was assessed using questionnaires and structured clinical interviews. Two groups of participants with prevailing NSSI and ceased NSSI were compared concerning their current psychological impairment, history of NSSI, suicide attempts, and BPD diagnosis.

**Results:**

Around half of all participants had engaged in NSSI within the last year, and around half met diagnostic criteria for BPD. Although there was no significant association between current NSSI and BPD, an earlier age of onset of NSSI and a longer duration of NSSI during adolescence was significantly predictive of adult BPD. Two thirds of participants still met criteria of an axis 1 psychiatric disorder. Suicide attempts were reported by 53.8 % of all participants. Participants with current NSSI were more likely to meet criteria for a current axis 1 disorder, had engaged in NSSI more often in their lifetime, and reported more suicide attempts.

**Conclusions:**

Reduction of NSSI from adolescence to young adulthood was lower than described in previous community samples. This may be due to the initial high psychiatric impairment of this sample in adolescence. Early onset of NSSI seemed to be a risk factor for a longer duration of NSSI during adolescence but not for NSSI prevailing into adulthood. However, it was a risk factor for adult BPD. Furthermore, the occurrence of suicidal thoughts and behaviors and prevailing NSSI was highly associated.

## Background

Non-suicidal self-injury (NSSI) is defined as the deliberate destruction of body tissue without suicidal intent [1]. Whereas NSSI is mentioned as a symptom of borderline personality disorder (BPD), especially in adolescence, it most often exists comorbid to further psychopathology like depressive or anxiety symptoms [2, 3]. However, it can also occur without further psychopathology [4, 5]. Recent prevalence studies have pointed towards NSSI being of major concern among adolescents, with international lifetime prevalence rates of around 18 % in the general population [6] and around 50 % in psychiatric in-patient samples [7].

A decline of prevalence rates of deliberate self-harm from late adolescence to young adulthood was shown in one large longitudinal community-based study with 1943 participants followed up in seven waves from age 15 to age 29 [8]. Naturally, only a small percentage (8 %) had engaged in self-harm at baseline and throughout the course of the study. Self-harm in young adulthood was significantly correlated with symptoms of anxiety and depression in adolescence.

In addition, a recent systematic review of longitudinal studies with sample sizes ranging from 49 to 936,449 participants provided evidence that NSSI seems to peak around the age of 16 years and decline throughout young adulthood [9]. Previous NSSI, depression, female gender, suicidality and psychological distress were among the most cited predictors for NSSI in the reviewed longitudinal studies. However, general assumptions about the longitudinal course of NSSI are not easily made, as studies included in this review were heterogenic regarding definitions of NSSI and its assessment, as well as timespans of follow-up periods and sample sizes.

Even though NSSI seems to decline in young adulthood, it has been shown to be a significant risk factor for suicide attempts in adolescents [2, 10, 11] and adults [12]. However, to our knowledge, only two studies have investigated the predictive nature of adolescent NSSI and suicide attempts in young adulthood: Tuisku et al. [13] found NSSI to be a significant predictor for suicide attempts at the 1 year follow-up (with participants being around 17.5 years of age), and at the 8-year follow-up. The Pittsburgh Girls Study collected data on NSSI, suicidal ideation and suicide attempts between ages 13 and 21, showing that a combination of suicidal ideation and NSSI increases the likelihood of a suicide attempt later on in life [14]. These findings seem to suggest that even though NSSI may decline over time, psychopathology tends to prevail.

As NSSI is mentioned as a symptom of BPD, and BPD usually also becomes apparent during adolescence [15], numerous studies assessed the link between the two. One of the first studies in a clinical sample of 89 adolescent psychiatric patients with recent NSSI reported a BPD prevalence of 51.7 % [16]. Interestingly, the exact same percentage (51.7 %) of BPD was found in a more recent study in adolescent psychiatric patients (*N* = 198) who fulfilled criteria for NSSI disorder according to DSM-5 [2]. In a recent study of 36 adolescents and young adults from an outpatient psychotherapy clinic, BPD affective dysregulation symptoms were more associated with intrapersonal functions of NSSI. The same was true for interpersonal BPD symptoms [17].

Given the high clinical relevance of NSSI as comorbid factor in psychiatric disorders and its role as risk factor for suicidality, there seems to be a gap in the literature regarding the longitudinal course of NSSI. This especially holds true for adolescents with severe NSSI and comorbid psychiatric disorders, as most longitudinal research stems from community samples. To the best of our knowledge, no study so far has followed-up patients, who were treated in child and adolescent psychiatry for NSSI in their adolescence into young adulthood.

The objectives of this study were to assess psychosocial functioning, suicidality, current NSSI and BPD in young adults, who had presented with NSSI to departments for child and adolescent psychiatry and psychotherapy in their past.

## Methods

The study was approved by the Institutional Review Board (IRB) of the University of Ulm and was carried out in accordance with the Declaration of Helsinki.

### Recruitment process

This study was conducted with former patients of the departments of child and adolescent psychiatry and psychotherapy in Ulm and Ravensburg, Germany. In order to recruit participants, archived files of every patient who had ever been treated at those two services in the years between October 2001 (foundation date of the Department of child and adolescent psychiatry and psychotherapy in Ulm) and December 2013 (end of data collection), and was now between 18 and 30 years old, were scanned electronically for NSSI related search terms (German wording for NSSI, self-injury, self-injurious behavior, self-mutilation, ICD-10 X78). Patients, in whose files at least one episode of NSSI had been recorded, were contacted. This procedure did not rely on self-reported identification of patients, but instead assured a clinically verified record of NSSI in medical reports, which is rather unique in NSSI research. We excluded participants who were currently still treated at one of the two services, and participants with insufficient knowledge of German language. However, participants with current ongoing psychotherapeutic/psychiatric treatment elsewhere were included in this study. Recruitment process took place in three separate waves over a time span of 2.5 years. Both, Ulm and Ravensburg, are mid-sized towns, surrounded by rural areas in the south of Germany. All participants, except for one participant with Turkish parents, were born in Germany.

In total, *N* = 545 former patients were identified and contacted by mail. The letter informed the potential participants about the purpose of the study. If they were willing to participate, they were asked to give written consent, to fill in and to return (via a prepaid envelope) a number of questionnaires, which had been sent out with the letter. No incentive was offered for returning questionnaires. Participants were also asked to state whether they would be willing to participate in an interview, and in this case to provide their phone number or email address. If participants were interested, they were invited for a semi-structured interview, which took place in Ulm or in Ravensburg. As an incentive for the interview, 25€ were offered. In case of no reply, addresses were updated using the official register of residents. However, due to legal regulations, only addresses of participants who still lived in the area of Ulm or Ravensburg could be retrieved.

### Materials

Participants gave information about general demographics and stated if they were in current psychotherapeutic or psychiatric treatment. Clinical axis 1 diagnoses, obtained at initial presentation during adolescence, were retrieved from clinical files.

General psychological impairment was assessed by the *Brief symptom inventory* (BSI, [18]), a 53-Item self-report questionnaire, using a 5-point Likert scale. The Global Severity Index (GSI) indicates overall psychological distress. The clinical cut-off is at GSI = 0.59, *T* = 61. Present Axis 1 diagnosis were assessed using the *Diagnostic Interview for Mental Disorders*–*short version* (Mini-DIPS, [19]). The Mini-DIPS is a well-established structured interview which assesses axis 1 disorders according to DSM-IV. All comorbid diagnoses were recorded.

As affective disorders were the most common diagnoses in the clinical files of patients at an adolescent age, we decided to add another instrument for assessing current depressive symptoms (*Beck Depression Inventory*, *second edition* (BDI-II; [20]; German version: [21])). The BDI-II assess DSM-IV criteria of major depression in 21 items. Results are classified as minimal depression (0–13), mild depression (14–19), moderate depression (20–28) and severe depression (29–63) [21]. Personality disorders were diagnosed by using the well-established Structured Clinical Interview for DSM-IV Axis II Personality Disorders (SCID-II) [22]. As the association of NSSI and BPD was of most interest, only data from group comparisons of current NSSI and BPD, and no other personality disorders, are presented in this paper. The semi-structured *Self*-*injurious thoughts and behaviors interview* (SITBI; [23]) was used to gain detailed information about participants’ NSSI and suicide attempts (present and lifetime). The interview consists of 169 items regarding frequency, functions and other characteristics of several self-injurious thoughts and behaviors. The German version (SITBI-G) showed good interrater reliability (*K* = 0.77) and construct validity (*K* = 0.89) scores for the assessment of NSSI in an adolescent sample of psychiatric inpatients [24]. To our knowledge, the SITBI has only been used in adolescent samples so far [23–25]. Since our study consisted of young adults only, comparable values of quality criteria can be expected. All interviews were conducted by trained clinical psychologists at master level or board certified child and adolescent child psychiatrists.

### Statistical analysis

Statistical analyses were carried out using IBM SPSS Statistics 21. Differences between groups were examined using Chi^2^-tests and *t*-tests. In cases where data was not normally distributed, Mann–Whitney-U-tests were applied. Alpha levels were Bonferroni-corrected. For all significant results, effect sizes were calculated (Cohen’s d for *t*-tests, *r*-values for Chi^2^ and Mann–Whitney-U-tests. *R*-values were then transformed into Cohen’s *d*-values, to make data comparable).

## Results

Of the *N* = 545 former patients who were contacted, *N* = 11 refused participation. *N* = 33 patients had moved and their current addresses could not be retrieved. *N* = 430 patients did not reply to the letter, although their addresses were up to date or had been updated. Of the *N* = 71 participants who returned completed questionnaires, *N* = 52 participated in an interview. Data of those 52 participants is presented in this paper. Socio-demographic data of participants who declined participation cannot be presented due to local IRB regulations.

### Socio-demographic characteristics of participants

All participants had reported at least one episode of NSSI prior to or during their psychotherapeutic/psychiatric treatment. On average, participants had been discharged from the ward 4.8 years (SD = 2.7) prior to the interview.

At the time of the interview, participants were on average *M* = 21.50 (SD = 2.61; min = 18, max = 28) years old. The majority of participants were female (94.2 %, *N* = 49). All participants had completed schooling. Secondary school was finished by 23.1 % (*N* = 12) after 9 years (German “Hauptschule”), 42.3 % (*N* = 22) after 10 years (“Realschule”) and 34.6 % (*N* = 18) after 13 years of schooling (“Gymnasium”). At the time of evaluation, 50 % (*N* = 26) of participants were currently studying or in an apprenticeship, 27 % (*N* = 14) were employed and 19.2 % (*N* = 10) were unemployed. Most participants did not have children (88.5 %, *N* = 46), three participants (5.8 %) had one child and one participant (1.9 %) had two children. Around half (53.8 %, *N* = 28) of participants were single, 32.7 % (*N* = 17) were in a relationship and 9.6 % (*N* = 5) were married.

### Psychological impairment

Around one third of participants (36.5 %, *N* = 19) reported being in current psychotherapeutic treatment and 30.8 % (*N* = 16) reported taking psychotropic medication regularly. However, 53.8 % (*N* = 28) of all participants reported neither being in psychotherapeutic treatment nor to be taking psychotropic medication.

The average Global Severity Index of the BSI showed a clinically significant score across all participants (M-GSI = 0.93, SD = 0.59; *T* = 71; min = 0.09, max = 2.51). However, 33 % (*N* = 17) of participants did not score in the clinically significant range (GSI ≤ 0.59, *T* ≤ 60). The average score of those participants who scored in a clinically significant range was at M-GSI = 1.17 (SD = 0.53), *T* = 74, which corresponds to the 99th percentile.

Concerning depressive symptoms, participants on average scored in a mild depressive range (M-BDI = 17.3, SD = 11.98; Median = 16.00; min = 1, max = 59). In detail, 36.5 % (*N* = 19) scored in a minimal range, 51.9 % (*N* = 27) scored in a mild depressive range, 13.5 % (*N* = 7) scored in a moderate range, and 15.4 % (*N* = 8) scored in a severely depressive range.

As adolescents, most participants had been diagnosed with major depression (see Table 1).

**Table 1:** Axis 1 diagnoses of participants

Axis 1 diagnosis	As adolescents		As young adults		Retainment	
	*N*	%	*N*	%	*N*	Percent of adolescent diagnosis
Major depression	31	59.6	20	38.5	15	48.4
Anxiety disorder	8	15.4	20	38.5	6	75.0
Adaptive disorder	12	32.1	-	-	-	-
Posttraumatic stress disorder	3	5.8	13	25	1	33.3
Eating disorder	9	17.3	13	25	7	77.8
Hyperkinetic disorder	3	5.8	-	-	-	-
Conduct disorder	8	15.4	-	-	-	-
Alcohol/drug abuse	4	7.7	8	15.4	2	50.0
Somatoform disorder	-	-	12	32.1	-	-
Obsessive compulsive disorder	-	-	4	7.7	-	-
Axis 2 diagnosis						
Antisocial PD	-	-	4	7.7	-	-
Avoidant PD	-	-	9	17.3	-	-
Borderline PD	-	-	24	46.2	-	-
Dependent PD	-	-	3	5.8	-	-
Depressive PD	-	-	10	19.2	-	-
Histrionic PD	-	-	1	1.9	-	-
Narcissistic PD	-	-	5	9.6	-	-
Negativistic PD	-	-	8	15.4		
Obsessive-Compulsive PD	-	-	9	17.3	-	-
Paranoid PD	-	-	7	13.5	-	-
Schizoid PD	-	-	0	0	-	-
Schizotypical PD	-	-	0	0	-	-

Numbers exceeding 100 % are due to comorbid diagnoses

*PD* personality disorder

According to the MINI-DIPS, 28.8 % (*N* = 15) of participants did not meet a current axis 1 psychiatric disorder. In more detail, 30.8 % (*N* = 16) met criteria for one, 21.2 % (*N* = 11) for two, 13.5 % (*N* = 7) for three and 5.8 % (*N* = 3) for four current axis 1 psychiatric disorders. The most common diagnoses were current episodes of major depression and anxiety disorders (specific phobia were excluded from analyses) (38.5 %, *N* = 20 respectively; for details see Table 1). A correlation analysis of clinical diagnoses in adolescence and at present revealed the following significant (alpha <0.003 Bonferroni corrected) intercorrelations: anxiety disorders in adolescence were significantly correlated with somatoform disorders in young adulthood (*r* = 0.40, *p* = 0.003), as well as conduct disorders in adolescence with substance dependence in young adulthood (*r* = 0.56, *p* < 0.001). Anxiety disorders (75 %, *N* = 6) and eating disorders (77.8 %, *N* = 7) had a high rate of retainment from adolescence to young adulthood.

Over half of all participants (61.5 %, *N* = 32) were diagnosed with a personality disorder according to the SCID-II. The most common diagnosis was borderline personality disorder (*N* = 24, 46.2 %), followed by depressive personality disorder (*N* = 10, 19.2 %, see Table 1).

### Suicidal behaviors

Out of all participants, 96.2 % (*N* = 50) reported having had suicidal thoughts in their lifetime, and 38.5 % (*N* = 20) reported having had suicidal thoughts within the last year. With regard to suicide attempts, 57.1 % (*N* = 28) of participants reported having attempted suicide at least once in their lifetime and 9.6 % (*N* = 5) reported having attempted suicide within the last year.

### Non-suicidal self-injury

On average, participants stated a lifetime number of NSSI of *M* = 334.46 episodes (SD = 576.80; min = 1, max = 2000). The median number of NSSI was *m* = 50.0. The average age of onset was 13.87 years (SD = 1.80; min = 9, max = 17). The most common method of NSSI was cutting (*N* = 48, 92.3 %), followed by scraping (*N* = 27, 51.9 %), and manipulating a wound (*N* = 22, 42.3 %). While 23.1 % (*N* = 12) of participants had used only one method of NSSI, the majority had used multiple methods (*M* = 3.21, SD = 2.07, min = 1, max = 10).

In total, 53.8 % (*N* = 28) of all participants had discontinued NSSI at least 1 year prior to the interview. Furthermore, 9.6 % (*N* = 5) of participants stated having thought about engaging in NSSI, but had been able to resist. Of those participants who had discontinued NSSI (at least within the last year), the average age of cessation was at *M* = 16.93 years (SD = 1.86, min = 14, max = 23).

Of the 46.2 % (*N* = 24) participants with prevailing NSSI, 41.7 % (*N* = 10) reported repetitive NSSI (on five or more occasions) within the last year. Out of those participants who had continued engaging in NSSI within the last year, the average number of episodes was *M* = 9.96 (SD = 13.51) times (median number of episodes = 3.50, min = 1, max = 50).

### Comparison of participants with and without prevailing NSSI

Participants who reported at least one incident of NSSI within the last year (*N* = 24) were compared to those who had ceased from NSSI for at least 1 year prior to the interview (*N* = 28).

#### Socio-demographic characteristics

There was no significant difference in current age between participants who had discontinued NSSI and those with prevailing NSSI (*p* = 0.34, *T* = 0.959). Furthermore, there were no statistically significant differences in participants who had continued or discontinued NSSI regarding their level of school education, employment status, and relationship status (see Table 2).

**Table 2:** Differences between young adults with and without prevailing NSSI

	Prevailing NSSI	Ceased NSSI				
T-tests	M (SD)	M (SD)	df	T	*p*	d
Current age	21.1 (2.5)	21.8 (2.7)	50	0.96	0.34	-
BSI-GSI	1.2 (0.63)	0.70 (0.44)	49	4.02	0.002	1.1
BDI-II	23.6 (12.4)	11.7 (8.4)	49	3.33	<0.001	1.3
Duration of NSSI (years)	7.2 (3.5)	3.0 (2.2)	50	5.26	<0.001	1.5
Age of onset NSSI	13.8 (1.9)	14.0 (1.7)	50	0.42	0.67	-
Mann–Whitney-U-Tests	rank-sum	rank-sum		Z	*p*	d
Frequency NSSI (lifetime)	775.5	602.5		2.57	0.010	0.77
Frequency suicide attempts (lifetime)	761.0	617.0		2.43	0.015	0.72
Frequency suicide attempts (last year)	681.0	697.0		1.62	0.106	-
Chi^2^-tests	*N* (%)	*N* (%)	df	Chi^2^	*p*	d
Single	16 (69.6)	12 (44.4)	1	3.18	0.093	-
Employed	19 (82.6)	21 (78.8)	1	0.181	0.736	-
Highest possible school education (Abitur)	8 (33.3)	10 (35.7)	1	0.032	1.00	-
Current psychotherapeutic treatment	15 (62.5)	4 (14.3)	1	12.96	<0.001	1.2
Current psychotropic medication	14 (58.3)	2 (7.1)	1	15.90	<0.001	1.3
Current Axis 1 disorder (Mini-DIPS)	23 (95.8)	14 (50.0)	1	13.23	<0.001	1.2
Current Major Depression	18 (75.0)	2 (7.1)	1	25.14	<0.001	1.9
Boderline personality disorder	12 (50)	12 (50)	1	0.265	0.781	-

Significance level of *p* < 0.05 Bonferroni-corrected: significance *p* < 0.003

#### Psychological impairment

Participants with prevailing NSSI were significantly (*p* < 0.001, Bonferroni corrected) more likely to receive psychotherapeutic treatment (62.5 % (*N* = 15) vs. 14.3 % (*N* = 4); *d* = 1.2) and to be taking psychotropic medication (58.3 % (*N* = 14) vs. 7.1 % (*N* = 2), *d* = 1.3).

Concerning self-report questionnaires, participants with prevailing NSSI scored significantly higher in the BSI (*p* = 0.002, *d* = 0.93), and the BDI-II (*p* < 0.001, *d* = 1.1).

Participants with prevailing NSSI were significantly more likely to be diagnosed with any axis-1 disorder according to DSM-IV (95.8 % (*N* = 23) vs. 50 % (*N* = 14); *p* < 0.001, *d* = 1.2). Also, they were diagnosed significantly more often with depressive disorders than participants who had stopped NSSI (75 % (*N* = 18) vs. 7.1 % (*N* = 2), *p* < 0.001, *d* = 1.9). There were no differences with regard to other psychiatric diagnoses (see Table 2).

#### Suicidal behaviors and NSSI

Regarding suicide attempts, participants with prevailing NSSI reported significantly more suicide attempts within their lifetime (*p* = 0.015, *d* = 0.72), but not significantly more attempts within the last year.

In addition, participants with prevailing NSSI reported significantly more episodes of NSSI in their lifetime (*p* = 0.010, *d* = 0.7). Furthermore, they had engaged in NSSI for a significantly longer time (*p* < 0.001, *d* = 1.5). There were no statistically significant differences between the two groups with regard to age of onset of NSSI or number of methods used (see Table 2).

### Relationship of characteristics in adolescence and young adulthood and the course of NSSI

There were no statistically significant correlations of any psychiatric diagnoses in adolescence and the duration, frequency or cessation of NSSI up to the current age of participants.

**Fig. 1:**
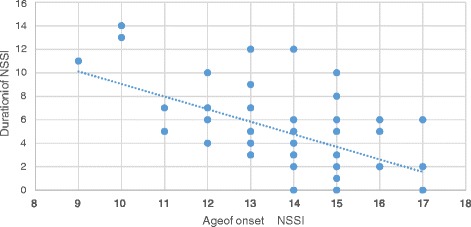
Duration of NSSI in association with age of onset of NSSI

However, a younger age of onset of NSSI was significantly correlated with a longer duration of NSSI (corrected for current age, *p* < 0.001, *r* = −0.73, see Fig. 1). However, it was not correlated with a higher frequency of NSSI or current NSSI.

The duration of NSSI in years was significantly correlated with the frequency of NSSI (lifetime), the number of suicide plans (lifetime), current BDI-II scores, and the number of current axis 1 diagnoses (see Table 3).

**Table 3:** Correlation of duration of NSSI with other characteristics

	Duration of NSSI (years)		
Pearson correlation (2-tailed)	*N*	*r*	*p*
Current age	52	0.34	0.013
Age of onset NSSI	52	−0.55	<0.001
Age of cessation NSSI	52	0.86	<0.001
Frequency NSSI (lifetime)	52	0.46	0.001
Frequency NSSI (last year)	52	0.37	0.007
Frequency suicide plans (lifetime)	52	0.41	0.002
Frequency suicide attempts (lifetime)	52	0.39	0.004
BSI-GSI	51	0.31	0.026
BDI-II	51	0.42	0.002
Number axis 1 diagnoses (Mini-DIPS)	52	0.40	0.003
Social support by at least one adult during childhood/adolescence	52	−0.30	0.033
Current psychotherapeutic treatment	52	0.23	0.108
Current psychotropic medication	52	0.34	0.014
Boderline Personality Disorder	52	0.43	0.001

Pearson correlation, *p* < 0.05 Bonferroni-corrected: significance *p* < 0.003

### Relationship of NSSI and borderline personality disorder (BPD)

There was no observed relationship between current NSSI and current diagnosis of BPD (Chi = 0.265, *p* = 0.78, see Table 2). Of those participants currently diagnosed with BPD (*N* = 24, 46.2 %), exactly 50 % (*N* = 12) reported NSSI within the last year, while the other 50 % did not (see Table 2). Of those participants without BPD (*N* = 28, 53.8 %), 42.9 % (*N* = 12) reported current NSSI, while 57.1 % (*N* = 16) did not.

However, there was a significant association (*Z* = 2.699, *p* = 0.007, *d* = 0.82) between the age of onset of NSSI and a current diagnosis of BPD. Participants with BPD had started NSSI significantly earlier in life (*M* = 13.12 years, SD = 2.01) than adolescents without BPD (*M* = 14.50 years, SD = 1.35). A similar association was found between BPD and duration of NSSI (*Z* = 2.93, *p* = 0.003, *d* = 0.94), Participants with BPD had engaged in NSSI for a significantly longer time (*M* = 6.54 years, SD = 3.71) than participants without BPD (*M* = 3.50 years, SD = 2.76) as was also shown by a positive correlation of BPD and duration of NSSI (*r* = 0.43, *p* = 0.001, see Table 3).

## Discussion

In this naturalistic follow-up study, former adolescent psychiatric patients, who had engaged in self-injury, were examined with regards to their current psychosocial functioning and psychopathology as young adults. Results of this study showed a weaker decline of NSSI from adolescence to young adulthood as has been shown in studies of the general population, with around 50 % still engaging in NSSI. Participants in this study generally still reported high psychiatric impairment. Prevailing NSSI was associated with greater current psychiatric impairment and a higher risk for suicide ideation. Although an early onset of NSSI did not seem to be a predictor for prevailing NSSI in young adulthood, it was significantly correlated to a longer duration of NSSI during adolescence and BPD in young adulthood.

### Psychiatric impairment

At assessment, only around one third of all participants were free of general psychopathology (as defined by not meeting clinically significant scores in self-assessment tools) and did not meet current axis 1 diagnoses in a clinical structured interview. The rate of current axis 1 diagnoses of around 70 % in this sample is by far higher than the 25–30 % estimated 12-month prevalence in the general population [26]. This may be explained by the fact that this is a very selected population as the initial sample consisted of highly impaired former adolescent psychiatric in-patients. It is therefore on the other hand more than noteworthy that 30 % did not meet any diagnosis anymore. At the same time, 53.8 % of participants did not take psychotropic medication and were not treated by a psychotherapist anymore. This could either point to the fact that around 20 % of participants are coping without treatment despite an axis 1 disorder, or to some percentage of participants not receiving professional help despite being in need for it. Non-treatment could furthermore point to the fact that there is a gap between child and adolescent and adult systems of psychiatric care in Germany, often leaving patients lost during their transition from childhood to adolescence [27].

### Socio-demographic characteristics

The level of education was slightly lower than in the general population aged 20–25 (23.1 vs. 17.2 % “Hauptschule”, 42.3 vs. 30.3 % “Realschule”, and 34.6 vs. 46.2 % Gymnasium) [28]. Furthermore, the unemployment rate of 20 % in this sample was also higher than the rate of around 6.7 % in the general population [29]. Conclusions of whether adolescent psychiatric impairment is associated with educational underachievement and higher unemployment rates cannot be drawn from this small sample, especially when taking the participation rate of 10 % into account. Rather, results may have been biased by favoring participation of unemployed adults who had more time at hand or were more drawn by the monetary incentive. However, results are in line with current research. Fergusson and Woodward [30] have shown a significantly increased risk for later educational underachievement and unemployment in adolescents with a major depression in a longitudinal study with 1265 participants aged 14–16 at baseline.

### Course of NSSI

Around half of all participants (46.2 %) still reported at least one episode of NSSI within the last year. Surprisingly, there was no age difference between participants who had ceased from engaging in NSSI and those who still self-injured. It has to be taken in account though, that with an average age of 21.5 years, this sample was still quite young. According to the literature [8, 9], it would have been expected for a larger number of participants to have ceased from NSSI by the age of 21. Again though, the low participation rate may have favored participants with prevailing NSSI to take part in the study. Furthermore, the population was not community based but consisted of individuals who had self-injured in a degree demanding inpatient care in their adolescence. It would be of great interest to conduct a long-term follow-up with these participants to investigate their long-term development.

Of those participants who had stopped engaging in NSSI, the average age of cessation was at 16.9 years. This is in accordance with Moran et al. [8] who found a significant drop of rates of self-harm between 15.9 and 17.4 years of age.

### NSSI and suicidality

In accordance with previous findings (i.e. [2, 12]) NSSI and suicidal thoughts and behaviors were strongly interlinked. Almost all (96.2 %) participants (who had all engaged in NSSI in their lives) reported suicidal thoughts in their lifetime and around half of them had tried to commit suicide at least once. Furthermore, in accordance with Tuisku et al. [13], those participants who still engaged in self-injury, reported more suicide attempts in their lives.

### Associative features of prevailing NSSI

In longitudinal studies, the most commonly described predictor for future NSSI has been previous NSSI [9]. Our findings add on to this outcome, since participants who still engaged in NSSI reported a longer duration and a higher frequency of NSSI throughout their lifetime. Interestingly, adolescents with an earlier onset of NSSI also reported a longer duration of NSSI in years during adolescence but not into their young adulthood. Therefore, early onset of NSSI might be a risk factor for a longer duration of NSSI throughout adolescence.

In a number of studies, depressive symptoms have been identified as predictor for NSSI [9]. Results of our study support a link between NSSI and depression, with participants still engaging in NSSI being significantly more likely to meet criteria of a current major depression and scored significantly higher in the BDI-II.

Participants with prevailing NSSI also scored significantly higher in the BSI, were more likely to be diagnosed with an axis 1 disorder, and were more likely to receive psychotherapeutic treatment or to be taking psychotropic medication. Prevailing NSSI from adolescence to young adulthood might therefore be a risk factor for overall psychological impairment. However, NSSI might also serve as a coping mechanism to deal with ongoing psychiatric symptoms.

### NSSI and BPD

We found a high prevalence rate of BPD in our sample of former adolescent psychiatric patients with NSSI. The rate of BPD diagnosis was 46.2 %, and slightly lower than the 51.7 % in previously investigated adolescent psychiatric samples with NSSI [2, 16]. Interestingly, in young adulthood, current NSSI was not associated with BPD diagnosis in our sample. However, adolescents with early onset of NSSI and long duration of NSSI during adolescence presented were at elevated risk to present with an adult diagnosis of BPD. The results may confirm the previously highlighted importance of developmental trajectories of NSSI and other self-harm behaviors as potential early markers of risk that may be used for early detection and intervention of BPD [15]. They may also well fit to a previous study indicating that first-presentation of BPD symptoms at young age could indicate a more severe prognosis of BPD [31].

### Limitations

Limitations of this study were a predominantly female sample, which did not allow to draw conclusions on possible gender differences. The overall small sample size disallowed further analyses (i.e. regression analyses). Furthermore, the low participation rate of around 10 % has to be taken into consideration, when interpreting results of this study. Therefore, results may have been biased by self-selection of participants (i.e. being interested in participating due to ongoing NSSI or declining participation due to high current psychiatric impairment). Further practical reasons (i.e. full-time employment, moving to other parts of Germany) may have led to declining participation. A number of possible participants could not be contacted, as due to legal privacy protection, current addresses could only be retrieved from participants who still lived in the state the study was conducted in. The majority of possible participants probably had moved away from their parents’ house after finishing school. Therefore, a low participation rate of former adolescent psychiatric inpatients is a commonly reported problem in follow-up studies [12]. In addition, data on lifetime NSSI and lifetime suicidal behavior was assessed retrospectively which may have distorted the results. However, all participants had been former psychiatric inpatients, and former clinical diagnosis and medical evidence of NSSI were available from reliable electronic archive systems, avoiding adolescent NSSI status to rely on self-report only.

## Conclusions

Overall, results of this study suggest a slight decrease of NSSI from adolescence to young adulthood in people with high psychiatric impairment and NSSI in adolescence. Early onset of NSSI seems to be a risk factor for a longer duration of NSSI in adolescence and an adult diagnosis of BPD, but not for prevailing NSSI in young adulthood. Furthermore, prevailing NSSI in young adulthood is associated with a greater risk for depression, suicide ideation, and other psychological impairment.

This exploratory study is, to our knowledge, the first study to follow up highly impaired adolescent psychiatric patients with NSSI into their young adulthood. Especially the relationship of NSSI in adolescence and its association with BPD later on in life is under researched. Although results are limited due to the small sample size and low participation rate, first conclusions can be drawn towards an association of early onset of NSSI and higher risk BPD in young adulthood. Larger longitudinal studies with clinical as well as healthy control groups would be needed to further investigate this question in more detail.
